# *Alloexidiopsis* gen. nov., A Revision of Generic Delimitation in *Auriculariales* (*Basidiomycota*)

**DOI:** 10.3389/fmicb.2022.894641

**Published:** 2022-07-12

**Authors:** Shi-Liang Liu, Zi-Qi Shen, Qian-Zhu Li, Xiang-Yang Liu, Li-Wei Zhou

**Affiliations:** ^1^State Key Laboratory of Mycology, Institute of Microbiology, Chinese Academy of Sciences, Beijing, China; ^2^College of Life Sciences, University of Chinese Academy of Sciences, Beijing, China; ^3^School of Life Science, Liaoning University, Shenyang, China

**Keywords:** *Agaricomycetes*, *Auriculariaceae*, *Exidiopsis*, *Heteroradulum*, wood-inhabiting fungi, six new taxa

## Abstract

*Auriculariales* is a fungal order with highly diverse morphological traits of basidiomes, which partially leads to a poor understanding of its taxonomic system at the generic level. To identify our recently collected specimens of *Auriculariales* to a species level, we perform a comprehensive phylogenetic analysis of the generic relationships in *Auriculariales*. In association with morphological characteristics, a new genus *Alloexidiopsis* belonging to *Auriculariaceae* is erected with two new species, namely, *A. australiensis* and *A. schistacea*. Moreover, *Exidiopsis calcea* separated from the generic type *E. effusa* and *Heteroradulum niveum* and *H. yunnanense* recently inaccurately described as members of *Heteroradulum* are recovered in the clade of *Alloexidiopsis*. These three species are thus transferred to this new genus. One collection of *Exidiopsis grisea* also falls in the clade of *Alloexidiopsis*, whereas another collection of this species is separated far from *Alloexidiopsis* and *E. effusa*. Since we have no collection to confirm the species identity of *E. grisea*, its generic position is uncertain. The main taxonomic morphological differences among *Alloexidiopsis* and related corticioid genera in *Auriculariales* are summarized. A key to all the five accepted species of *Alloexidiopsis* is provided. As two unnamed lineages exist in *Alloexidiopsis* besides the abovementioned five species, it is assumed that more new species will be revealed from this genus under its current circumscription.

## Introduction

*Auriculariales* is a fungal order being mainly composed of wood-inhabiting macrofungi in *Agaricomycetes* and *Basidiomycota* (Hibbett et al., 2007). The type genus of this order is *Auricularia*, which together with several other gelatinous genera, namely, *Exidia, Guepinia*, and *Pseudohydnum*, comprise important edible and medicinal fungi (Wu et al., 2019). Therefore, interest in species diversity in these gelatinous genera has grown significantly in recent years (Bandara et al., 2015; Chen et al., 2020; Shen and Fan, 2020; Ye et al., 2020; Wang and Thorn, 2021; Wu et al., 2021).

Contrary to the gelatinous genera, most species in *Auriculariales* bear tough, resupinate, and effused to reflexed basidiomes as corticioid and polyporoid fungi (Miettinen et al., 2012; Zhou and Dai, 2013; Malysheva and Spirin, 2017). With the aid of molecular phylogeny, the corticioid species traditionally placed in *Eichleriella, Exidiopsis*, and *Heterochaete* according to morphological characters have been rearranged to make genera monophyletic. After the erection of some new genera, e.g., *Adustochaete, Amphistereum, Crystallodon, Proterochaete*, and *Sclerotrema* and reinstatement of several previously known genera, e.g., *Hirneolina, Heteroradulum*, and *Tremellochaete* (Malysheva and Spirin, 2017), *Eichleriella* is accepted to be a monophyletic genus, while *Exidiopsis* and *Heterochaete* seem to be synonymous with a priority of the latter genus (Malysheva and Spirin, 2017; Alvarenga et al., 2019; Alvarenga and Gibertoni, 2021). However, certain species of *Exidiopsis*, even sequenced ones such as *E. calcea* and *E. grisea*, still have no appropriate placement at the generic level (Malysheva and Spirin, 2017; Li et al., 2022b). In addition, the generic placement of certain recently described species of *Heteroradulum* is questionable as indicated in a study by Li et al. (2022b) and our understanding of the phylogenies in Guan et al. (2020) and Li et al. (2022a). This phenomenon indicates the generic delimitation in *Auriculariales* that should be further clarified.

When revisiting specimens collected in the last few years, some of them are identified to be previously known and new species in *Auriculariales*, but cannot be placed in any known genus. Therefore, a new genus is erected for these species and also for other related species.

## Materials and Methods

### Morphological Examination

Sixteen studied specimens were sampled in northwestern and southwestern China, Vietnam, and Australia from May to November 2017–2020. These specimens were dried using a portable drying instrument at 35°C on the day of sampling and are preserved at the Fungarium, Institute of Microbiology, Chinese Academy of Sciences (HMAS), Beijing, China and the National Herbarium of Victoria (MEL), Melbourne, Australia. Macromorphological characters of basidiomes were examined with the aid of a Leica M125 stereomicroscope (Wetzlar, Germany) at magnifications up to 100 × . Color terms follow Petersen (1996). Microscopic examination was carried out with an Olympus BX43 light microscope (Tokyo, Japan) at magnifications up to 1,000 × following a study by Liu et al. (2021). All the measurements were taken from the sections mounted in cotton blue. The following abbreviations are used: L = mean basidiospore length (arithmetic average of all the basidiospores), W = mean basidiospore width (arithmetic average of all the basidiospores), Q = variation in the L/W ratios between the specimens studied, and n = number of basidiospores measured from a given number of specimens.

### Deoxyribonucleic Acid Extraction and Sequencing

The cetyltrimethylammonium bromide (CTAB) plant genome rapid extraction kit (Beijing Demeter Biotech Co., Ltd., Beijing, China) was employed for DNA extraction from dried specimens. The internal transcribed spacer (ITS) and nuclear large subunit (nLSU) gene regions were amplified with the primer pairs ITS5/ITS4 (White et al., 1990) and LR0R/LR7 (Vilgalys and Hester, 1990), respectively. The PCR procedure for ITS was initial denaturation at 95°C for 3 min, followed by 35 cycles at 94°C for 40 s, 54°C for 45 s, 72°C for 1 min, and a final extension of 72°C for 10 min, while that for nLSU was initial denaturation at 94°C for 1 min, followed by 34 cycles at 94°C for 30 s, 50°C for 1 min, 72°C for 1.5 min, and a final extension of 72°C for 10 min. The PCR products were purified and sequenced at the Beijing Genomics Institute (BGI), China. All the newly generated sequences were submitted to GenBank (https://www.ncbi.nlm.nih.gov/genbank/).

### Phylogenetic Analysis

The current dataset for phylogenetic analysis included all the main lineages in *Auriculariales* as ingroup taxa, while *Sistotrema brinkmannii* was selected as an outgroup taxon following a study by Li et al. (2022b). The ITS and nLSU regions were separately aligned using MAFFT version 7.110 (Katoh and Standley, 2013) with the G-INS-i strategy (Katoh et al., 2005), and then the two resulting alignments were concatenated as a single alignment. The concatenated alignment was submitted to TreeBASE (http://www.treebase.org; accession number S29452). jModelTest 2.1.10 (Guindon and Gascuel, 2003; Darriba et al., 2012) was used to determine the best-fit evolutionary model of the concatenated alignment based on the Akaike information criterion (AIC). Following the resulting model, maximum likelihood (ML) and Bayesian inference (BI) analyses were performed. For the ML analysis, raxmlGUI 2.0 (Stamatakis, 2014; Edler et al., 2021) was used with the calculation of bootstrap (BS) replicates under the auto fiber channel (FC) option (Pattengale et al., 2009). For the BI analysis, MrBayes 3.2 (Ronquist et al., 2012) was used with two independent runs of four chains, and trees were sampled every 1,000th generation. The first 25% of the resulting trees were discarded as burn-in, while the remaining 75% of the resulting trees were used for constructing a 50% majority consensus tree and calculating Bayesian posterior probabilities (BPPs). Chain convergence was determined using Tracer 1.7 (Rambaut et al., 2018). The trees were visualized in FigTree 1.4.4 (Rambaut, 2018) and edited in Adobe Illustrator cc 2020.

## Results

**Figure 1:**
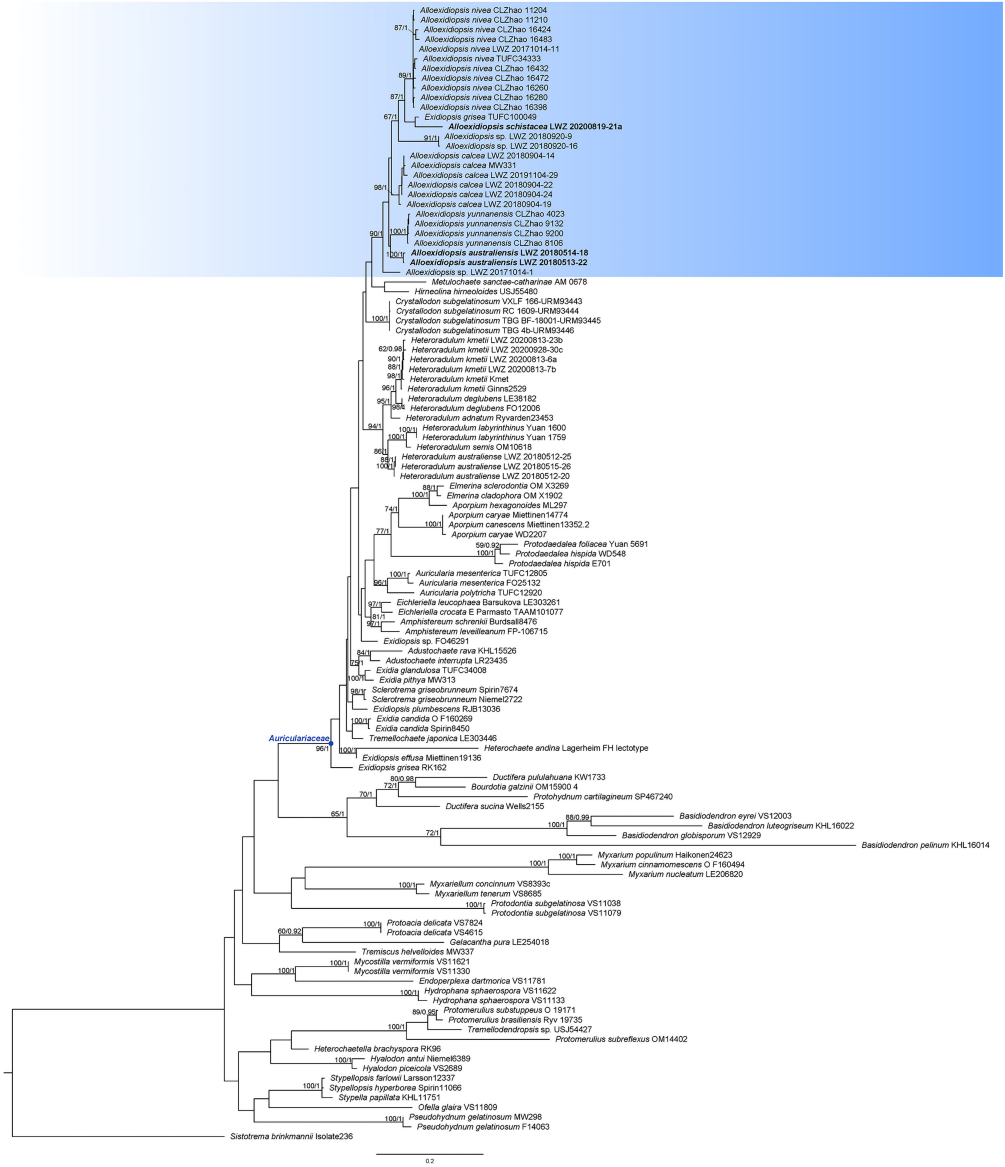
Phylogenetic position of *Alloexidiopsis* in *Auriculariales* inferred from the concatenated dataset of internal transcribed spacer (ITS) and nuclear large subunit (nLSU) regions. The topology generated from the maximum likelihood analysis is shown along with bootstrap values and Bayesian posterior probabilities of more than 50% and 0.8, respectively, at the nodes. The new genus *Alloexidiopsis* is highlighted with the bluish background color, while the specimens of the newly described species are in boldface.

A total of 15 ITS and 15 nLSU sequences were newly generated from all the 16 studied specimens (Table 1). The concatenated alignment of ITS and nLSU regions has 117 collections and 1,675 characters. GTR + I + G was estimated as the best-fit evolutionary model for this alignment. The ML analysis ended after 200 BS replicates. The BI analysis converged after 20 million generations, which was indicated by the effective sample sizes of all the parameters above 5,000 and the potential scale reduction factors close to 1.000. The topology resulting from the ML analysis is shown along with BS values of more than 50% and BPPs of more than 0.8 at the nodes (Figure 1).

**Table 1:** Species and sequences used in the phylogenetic analyses.

**Species**	**Voucher number**	**GenBank accession number**	
		**ITS**	**nLSU**
*Adustochaete rava*	KHL15526	MK391517	MK391526
*Adustochaete interrupta*	LR23435	MK391518	MK391527
*Alloexidiopsis australiensis*	LWZ 20180513-22	**OM801933**	**OM801918**
*A. australiensis*	LWZ 20180514-18	**OM801934**	**OM801919**
*A. calcea*	MW 331	AF291280	AF291326
*A. calcea*	LWZ 20180904-14	**OM801935**	**OM801920**
*A. calcea*	LWZ 20180904-19	**OM801936**	**OM801921**
*A. calcea*	LWZ 20180904-22		**OM801922**
*A. calcea*	LWZ 20180904-24	**OM801937**	**OM801923**
*A. calcea*	LWZ 20191104-29	**OM801938**	**OM801924**
*A. nivea*	CLZhao 11204	MZ352947	MZ352932
*A. nivea*	CLZhao 11210	MZ352948	MZ352933
*A. nivea*	CLZhao 16260	MZ352940	MZ352934
*A. nivea*	CLZhao 16280	MZ352941	MZ352935
*A. nivea*	CLZhao 16398	MZ352942	MZ352936
*A. nivea*	CLZhao 16424	MZ352943	MZ352937
*A. nivea*	CLZhao 16432	MZ352944	
*A. nivea*	CLZhao 16472	MZ352945	MZ352938
*A. nivea*	CLZhao 16483	MZ352946	MZ352939
*A. nivea*	LWZ 20171014-11	**OM801941**	**OM801926**
*A*. *nivea*	TUFC34333	AB871764	AB871745
*A. schistacea*	LWZ 20200819-21a	**OM801939**	**OM801932**
*A. yunnanensis*	CLZhao 4023	MT215568	MT215564
*A. yunnanensis*	CLZhao 8106	MT215569	MT215565
*A. yunnanensis*	CLZhao 9132	MT215570	MT215566
*A. yunnanensis*	CLZhao 9200	MT215571	MT215567
*A*. sp.	LWZ 20171014-1	**OM801940**	**OM801925**
*A*. sp.	LWZ 20180920-9	**OM801943**	**OM801928**
*A*. sp.	LWZ 20180920-16	**OM801942**	**OM801927**
*Amphistereum leveilleanum*	FP-106715	KX262119	KX262168
*A. schrenkii*	Burdsall 8476	KX262130	KX262178
*Aporpium canescens*	Miettinen 13352.2	JX044152	
*A. caryae*	Miettinen 14774	JX044145	
*A. caryae*	WD2207	AB871751	AB871730
*A. hexagonoides*	ML297	AB871754	AB871735
*Auricularia mesenterica*	FO 25132	AF291271	AF291292
*A. mesenterica*	TUFC12805	AB915192	AB915191
*A. polytricha*	TUFC12920	AB871752	AB871733
*Basidiodendron eyrei*	VS 12003	MT040880	MT040854
*B. globisporum*	VS 12929	MT040884	MT040864
*B. luteogriseum*	KHL 16022	MT040881	MT040861
*B. pelinum*	KHL 16014	MT040875	MT040862
*Bourdotia galzinii*	OM 15900.4	MG757511	MG757511
*Crystallodon subgelatinosum*	RC 1609-URM93444	MN475888	MN475884
*C. subgelatinosum*	TBG BF-18001-URM93445	MN475889	MN475885
*C. subgelatinosum*	TBG 4b-URM93446	MN475890	MN475886
*C. subgelatinosum*	VXLF 166-URM93443	MN475887	
*Ductifera pululahuana*	KW 1733		AF291315
*D. sucina*	Wells 2155	AY509551	AY509551
*Eichleriella crocata*	TAAM 101077	KX262100	KX262147
*E. leucophaea*	LE 303261	KX262111	KX262161
*Elmerina cladophora*	OM X1902	MG757509	MG757509
*E. sclerodontia*	OM X3269	MG757512	MG757512
*Endoperplexa dartmorica*	VS 11781	MT235621	MT235602
*Exidia candida*	O F160269	KY801872	KY801897
*E. candida*	Spirin 8450	KY801875	KY801900
*E. glandulosa*	TUFC34008	AB871761	AB871742
*E. pithya*	MW 313	AF291275	AF291321
*Exidiopsis effusa*	Miettinen 19136	KX262145	KX262193
*E. grisea*	RK 162	AF291281	AF291328
*E. grisea*	TUFC100049	AB871765	AB871746
*E. plumbescens*	RJB 13036	AF395309	AF395309
*E*. sp.	FO 46291	AF291282	AF291329
*Gelacantha pura*	LE 254018	MK098882	MK098930
*Heterochaete andina*	Lagerheim		KX262187
*Heterochaetella brachyspora*	RK 96		AF291337
*Heteroradulum adnatum*	Ryvarden23453	KX262116	KX262165
*H. australiense*	LWZ 20180512-20	MZ325254	MZ310424
*H. australiense*	LWZ 20180512-25	MZ325255	MZ310425
*H. australiense*	LWZ 20180515-26	MZ325256	MZ310426
*H. deglubens*	FO12006	AF291272	AF291318
*H. deglubens*	LE 38182	KX262112	KX262162
*H. labyrinthinus*	Yuan 1600	KM379139	KM379140
*H. labyrinthinus*	Yuan 1759	KM379137	KM379138
*H. kmetii*	Ginns 2529	KX262135	KX262183
*H. kmetii*	Kmet	KX262124	KX262173
*H. kmetii*	LWZ 20200813-6a	**OM801944**	**OM801929**
*H. kmetii*	LWZ 20200813-7b	**OM801945**	
*H. kmetii*	LWZ 20200813-23b	**OM801946**	**OM801930**
*H. kmetii*	LWZ 20200928-30c	**OM801947**	**OM801931**
*H. semis*	OM10618	KX262146	KX262194
*Hirneolina hirneoloides*	USJ 55480	AF291283	AF291334
*Hyalodon antui*	Niemelä 6389	MG735416	MG735424
*H. piceicola*	VS 2689	MG735414	MG735422
*Hydrophana sphaerospora*	VS 11133	MK098883	MK098931
*H. sphaerospora*	VS 11622	MK098884	MK098932
*Metulochaete sanctae-catharinae*	AM 0678	MK484065	MK480575
*Mycostilla vermiformis*	VS 11330	MG735417	MG735425
*M. vermiformis*	VS 11621	MG857093	MG857098
*Myxariellum concinnum*	VS 8393c	MK098885	MK098933
*M. tenerum*	VS 8685	MK098886	MK098934
*Myxarium cinnamomescens*	O F160494	KY801882	KY801909
*M. nucleatum*	LE 206820	KY801869	KY801894
*M. populinum*	Haikonen 24623	KY801883	KY801910
*Ofella glaira*	VS 11809	MK098920	MK098964
*Protoacia delicata*	VS 4615	MK098923	MK098967
*P. delicata*	VS 7824	MK098922	MK098966
*Protodaedalea foliacea*	Yuan 5691	JQ764666	JQ764644
*P. hispida*	E701	AB871767	AB871748
*P. hispida*	WD 548	AB871768	AB871749
*Protodontia subgelatinosa*	VS 11038	MK098926	MK098969
*P. subgelatinosa*	VS 11079	MG735412	MG735420
*Protohydnum cartilagineum*	SP467240	MG735419	MG735426
*Protomerulius brasiliensis*	Ryv.19735		AF291359
*P. subreflexus*	OM 14402	MG757508	
*P. substuppeus*	O 19171	JX134482	JQ764649
*Pseudohydnum gelatinosum*	F14063	AF384861	AF384861
*P. gelatinosum*	MW 298	DQ520094	
*Sclerotrema griseobrunneum*	Niemelä 2722	KX262144	KX262192
*S. griseobrunneum*	Spirin 7674	KX262140	KX262188
*Sistotrema brinkmannii*	Isolate 236	JX535169	JX535170
*Stypella papillata*	KHL 11751	EU118672	EU118672
*Stypellopsis farlowii*	Larsson 12337	MG857095	MG857099
*S. hyperborea*	Spirin 11066	MG857096	MG857102
*Tremellochaete japonica*	LE 303446	KX262110	KX262160
*Tremellodendropsis* sp.	USJ 54427		AF291375
*Tremiscus helvelloides*	MW 337		AF291377

*Newly generated sequences are in bold*.

The *Auriculariaceae* is well recovered (BS = 96%, BPP = 1) by the current phylogeny (Figure 1). In *Auriculariaceae*, besides four sequences representing *Heteroradulum kmetii* (LWZ 20200813-6a, LWZ 20200813-7b, LWZ 20200813-23b, and LWZ 20200928-30c), additional newly sequenced specimens (Table 1) grouped with *Exidiopsis calcea*, one of the collections of “*E. grisea*” (TUFC 100049), *Heteroradulum niveum*, and *H. yunnanense* as a strongly supported clade (BS = 94%, BPP = 1) that is separated from the generic types of *Exidiopsis* (*E. effusa*) and *Heteroradulum* (*H. kmetii*). This clade is described as a new genus below. In this clade, five of our new sequences turned out to represent *E. calcea* (LWZ 20180904-14, LWZ 20180904-19, LWZ 20180904-22, LWZ 20180904-24, and LWZ 20191104-29), one belongs to *H. niveum* (LWZ 20171014-11). The remaining sequences formed four new lineages. The specimens such as LWZ 20171014-1, LWZ 20180920-9, and LWZ 20180920-16 are sterile and thus, the two lineages represented by them are not included in the subsequent taxonomic treatment. The other two lineages, represented by the specimens LWZ 20180513-22 and LWZ 20180514-18 and LWZ 20200819-21a are, respectively, described as two new species in association with morphological examinations. *Exidiopsis calcea, H. niveum*, and *H. yunnanense* are transferred to the new genus, while the species identity of “*E. grisea*” cannot be confirmed and thus, a taxonomic change for this species at the generic level is not proposed.

### Taxonomy

***Alloexidiopsis*** L.W. Zhou & S.L. Liu, *gen. nov*.

MycoBank: MB 844125.

Etymology: *Alloexidiopsis* (Latin), refers to the segregation from *Exidiopsis*.

Diagnosis: It differs from *Exidiopsis* in the combination of resupinate, leathery basidiomes and the presence of cystidia and hyphidia.

Type species: *Alloexidiopsis schistacea* S.L. Liu, Z.Q. Shen & L.W. Zhou (described below).

Type specimen: **China:** Sichuan, Pingshan County, Laojunshan National Nature Reserve, on the fallen angiosperm trunk, 19 August 2020, *LW Zhou*, LWZ 20200819-21a (holotype in HMAS).

Description: Basidiomes annual, resupinate, effused, thin, leathery, closely adnate. Hymenophore smooth or with sterile spines, greyish white to ochraceous, cracked or not. Hyphal system monomitic, generative hyphae with clamp connections, hyaline, thin-walled. Cystidia cylindrical to clavate, thin-walled. Hyphidia abundant, covering hymenium, branched, thin-walled. Basidia ellipsoid to ovoid, longitudinally septate, two- to four-celled, hyaline. Basidiospores cylindrical to broadly cylindrical, slightly curved (allantoid), hyaline, thin-walled, smooth, inamyloid, indextrinoid, acyanophilous. On wood.

Notes: *Alloexidiopsis* is characterized by grayish-white to ochraceous, corticioid basidiomes, a monomitic hyphal system, and the presence of cystidia and hyphidia. Besides *Exidiopsis* as indicated in diagnosis, this new genus is also close to *Crystallodon* and *Heteroradulum* in morphology. However, *Crystallodon* differs in the presence of hyphal pegs surrounded by crystals (Alvarenga and Gibertoni, 2021), while *Heteroradulum* has brightly colored (pinkish or reddish) basidiomes and a mono- or dimitic hyphal system with thick-walled generative hyphae (Malysheva and Spirin, 2017; Li et al., 2022b). The main taxonomic morphological differences among *Alloexidiopsis* and related corticioid genera in *Auriculariales* are summarized in Table 2.

**Table 2:** Morphological comparison among *Alloexidiopsis* and related corticioid genera in *Auriculariales*.

**Genus**	**Basidiomes**	**Hymenophore**	**Hyphal system**	**Cystidia**	**Hyphidia**	**Basidiospores**
*Adustochaete*	Annual, small-sized, orbicular, waxy	Spiny or tuberculate, grayish to brownish	Monomitic	Clavate to fusiform, thin-walled	Variably branched	Cylindrical to broadly cylindrical, straight or curved
*Alloexidiopsis*	Annual, effused, leathery	Smooth or with sterile spines, more or less grayish	Monomitic	Cylindrical to clavate, thin-walled	Nodulose or richly branched	Cylindrical to broadly cylindrical, slightly curved
*Amphistereum*	Annual or perennial, cupulate-orbicular, hard leathery	Smooth, pale-colored	Dimitic	Rare, narrowly clavate, thin-walled	Richly branched	Cylindrical to broadly cylindrical, slightly curved
*Crystallodon*	Annual, effused, gelatinous to crustaceous	Covered by sharp-pointed sterile spines, brownish	Monomitic	Fusiform to cylindrical, often sinuous, thin-walled	Branched	Cylindrical to broadly cylindrical, slightly curved
*Exidiopsis* (*Heterochaete*)	Annual, effused or effused-reflexed, waxy gelatinous, arid waxy or coriaceous	Smooth or with sterile spines, gray, buff, ochraceous	Monomitic	Present or absent, cylindrical, clavate or fusiform, thin-walled	Simple or richly branched	Subglobose, ellipsoid, cylindrical to allantoid
*Heteroradulum*	Annual or perennial, effused-reflexed, leathery	Smooth, with sterile spines, pinkish or reddish	Mono- or dimitic	Clavate to fusiform, thin to thick-walled	Richly branched	Cylindrical to broadly cylindrical, sometimes curved
*Metulochaete*	Effused, gelatinous to waxy-arid	Smooth or covered by sterile spines, pale-colored	Monomitic	Metuloid, covering hymenial spines, thick-walled	Richly branched	Allantoid, straight to slightly curved
*Proterochaete*	Annual, orbicular, arid	Smooth or irregularly spiny, cream-colored to grayish or pale ochraceous	Monomitic	Occasional, sinuous, accidentally dichotomously branched, thin-walled	Richly or sparsely branched	Cylindrical to broadly cylindrical, slightly curved
*Sclerotrema*	Perennial, orbicular, leathery	Smooth, grayish brown	Monomitic	Hyphoid to fusiform, thick-walled	Richly branched	Allantoid, distinctly curved

**Figure 2:**
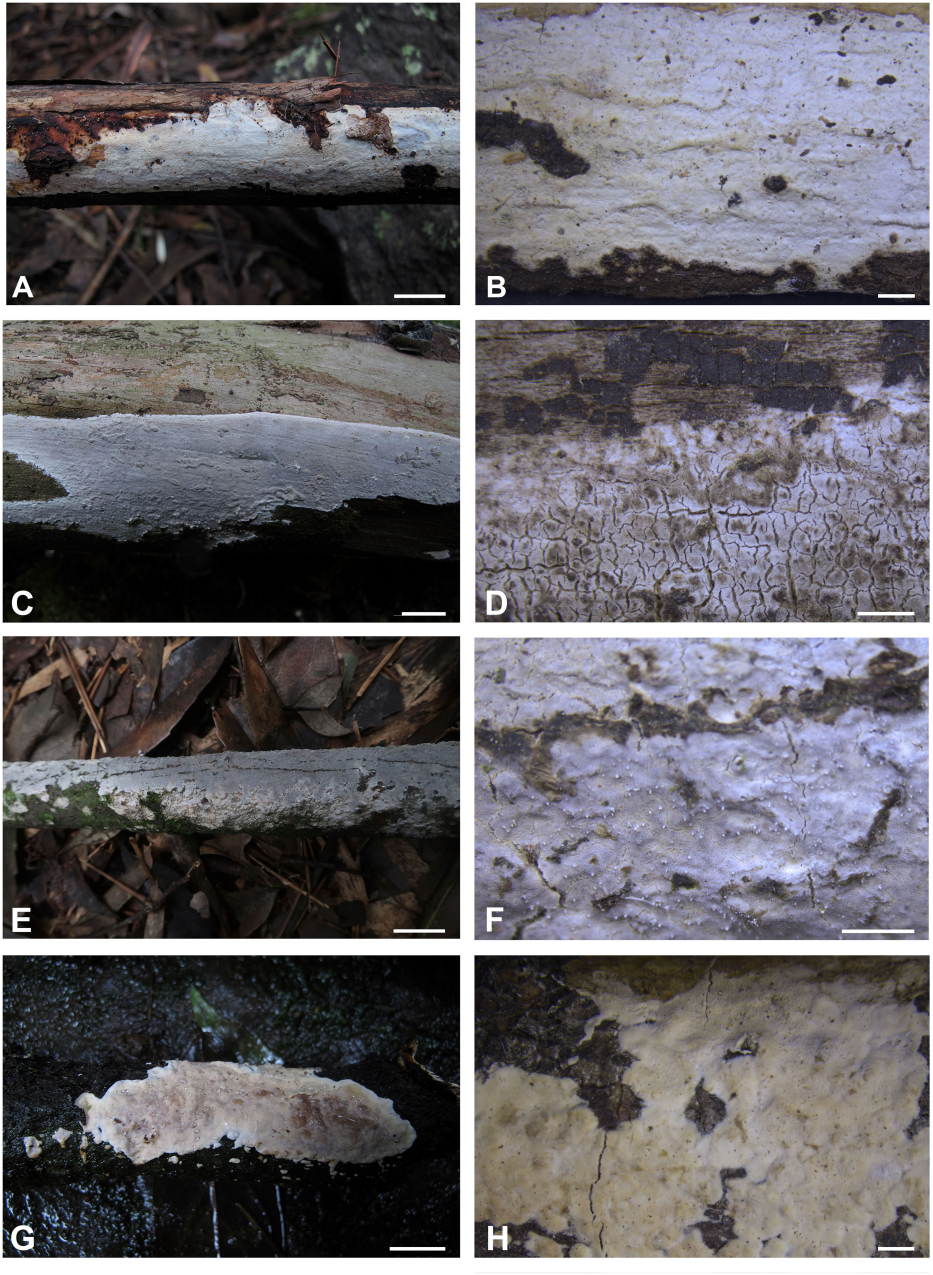
Basidiomes of *Alloexidiopsis*. **(A,B)** *A. australiensis* (LWZ 20180513-22, holotype). **(C,D)** *A. calcea* (LWZ 20180904-24). **(E,F)** *A. schistacea* (LWZ 20200819-21a, holotype). **(G,H)** *A*. sp. (LWZ 20180920-16). Scale bars: **(A,C,E,G)** = 1 cm, **(B,D,F,H)** = 2 mm.

**Figure 3:**
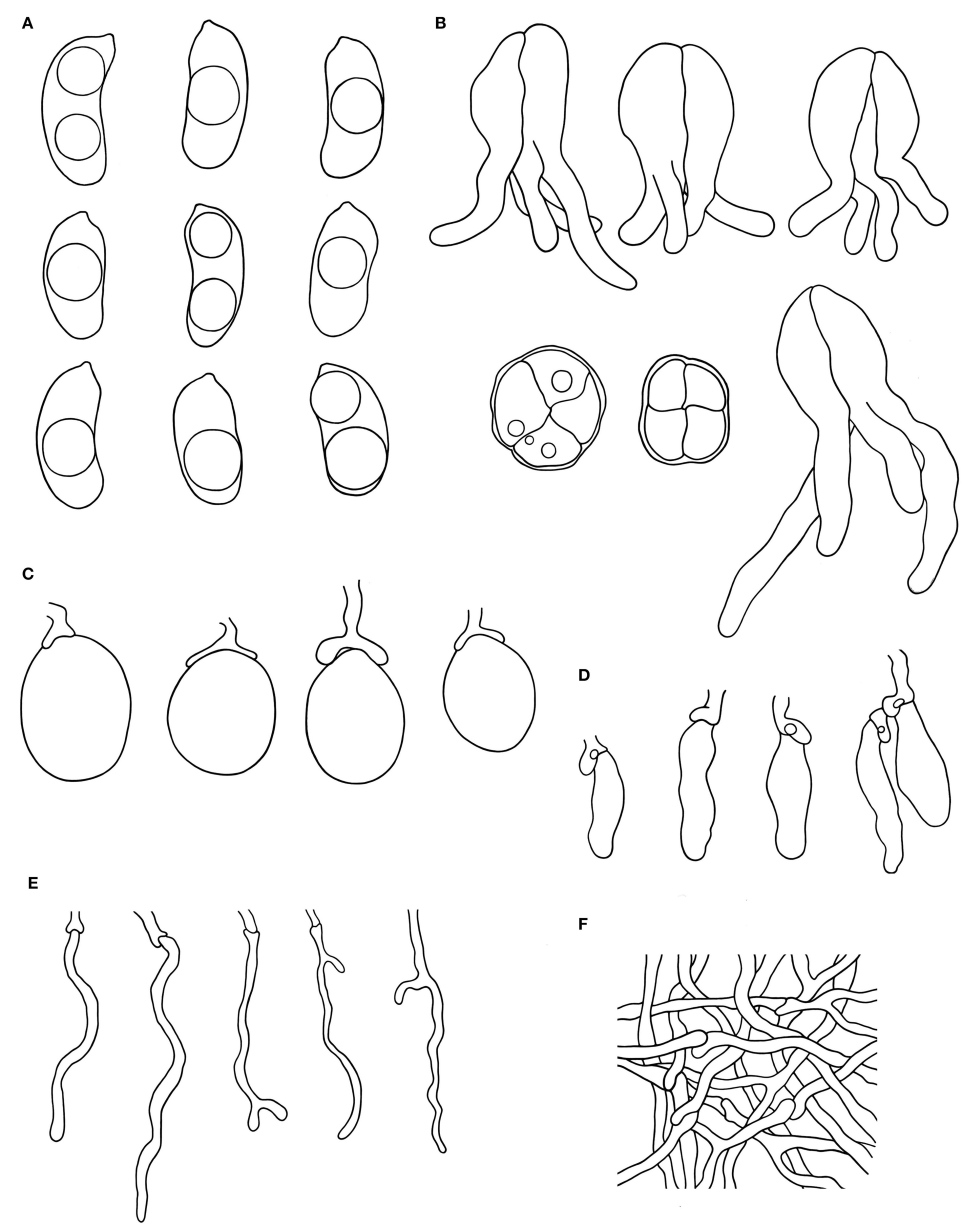
Microscopic structures of *Alloexidiopsis australiensis* (drawn from the holotype). **(A)** Basidiospores. **(B)** Basidia. **(C)** Basidioles. **(D)** Cystidia. **(E)** Hyphidia. **(F)** Hyphae from subiculum. Scale bars = 10 μm.

***Alloexidiopsis australiensis*** S.L. Liu, Z.Q. Shen & L.W. Zhou, *sp. nov*. (Figures 2A,B, 3).

MycoBank: MB 844126.

Etymology: *australiensis* (Latin), refers to Australia.

Diagnosis: It is characterized by smooth, cream hymenophore.

Type: **Australia:** Tasmania, Hobart, and Mount Wellington, on the fallen angiosperm branch, 13 May 2018, *LW Zhou*, LWZ 20180513-22 (holotype in MEL, isotype in HMAS).

Description: Basidiomes annual, resupinate, membranaceous, becoming leathery upon drying, closely adnate, widely effused, up to 12 cm long, 2 cm wide, 100–200 μm thick. Hymenophore smooth, cream to pale orange when fresh, becoming white upon drying. Margin gradually thinning out, thin, concolorous with or slightly darker than subiculum.

Hyphal system monomitic; generative hyphae with clamp connections. Subiculum composed of crystal clusters and agglutinated hyphae; subicular hyphae hyaline, thin-walled, frequently branched, closely interwoven, 1–2 μm in diam. Cystidia cylindrical with an obtuse apex, ventricose, 21.5–24.5 × 9.5–12 μm, with a clamp connection at base. Hyphidia arising from hyphae, nodulose or richly branched, hyaline, thin-walled, 22–33 × 1–2 μm. Basidia ellipsoid to ovoid, longitudinally septate, four-celled, embedded, 18–21 μ 13–18 μm, occasionally with a short base stalk, with a clamp connection at base. Basidiospores cylindrical to broadly cylindrical, slightly curved (allantoid), hyaline, thin-walled, smooth, acyanophilous, inamyloid, indextrinoid, with oily inclusions, (12−)13–25(−25.5) × (6.5−)7–11(−12) μm, *L* = 20.0 μm, *W* = 9.0 μm, *Q* = 2.3 (*n* = 60/2).

Other specimens (paratype) are also examined: **Australia:** Timbs Track, on dead standing angiosperm, 14 May 2018, *LW Zhou*, LWZ 20180514-18 (HMAS).

Notes: *Alloexidiopsis australiensis* resembles *A. calcea* and *A. nivea* (both transferred below) by smooth hymenophore in *Alloexidiopsis*. However, *A. calcea* differs in grayish-white to ochraceous hymenophore when fresh and has a distribution in the Northern Hemisphere (Wells, 1961), while *A. nivea* differs in smaller basidiospores (6.5–13.5 × 2.7–5.5 μm; Li et al., 2022a). *Exidiopsis macrospora* is similar to *A. australiensis* by the leathery basidiomes and the presence of cystidia and hyphidia; however, it differs in the reflexed basidiomes when dry and smaller basidiospores (10–15 μm × 5–7.5 μm; Wells, 1961).

***Alloexidiopsis calcea*** (Pers.) L.W. Zhou & S.L. Liu, *comb*. *nov*. (Figures 2C,D).

MycoBank: MB 844128.

*Basionym*: *Thelephora calcea* Pers., Syn. meth. fung. (Göttingen) 2:581 (1801).

≡ *Auricularia calcea* (Pers.) Mérat, Nouv. Fl. Environs Paris, Edn 2 1:35 (1821).

≡ *Corticium calceum* (Pers.) Fr., Epicr. syst. mycol. (Upsaliae): 562 (1838) (1836–1838).

≡ *Terana calcea* (Pers.) Kuntze, Revis. gen. pl. (Leipzig) 2:872 (1891).

≡ *Sebacina calcea* (Pers.) Bres., Fung. trident. 2(11–13):64 (1892).

≡ *Exidiopsis calcea* (Pers.) K. Wells, Mycologia 53(4):348 (1962) (1961).

Notes: *Alloexidiopsis calcea* has been successively placed in several genera. Before the current study, its latest generic placement was *Exidiopsis*, which is accepted by the first and also the only comprehensively phylogenetic analyses of *Auriculariales* (Weiß and Oberwinkler, 2001). The phylogeny in Malysheva and Spirin (2017) recognized that *Exidiopsis calcea* was separated from the generic type *E. effusa*, but no taxonomic change was proposed may be due to a lack of specimens for careful morphological examinations. Here, five additional specimens were collected from Northwest and Southwest China grouped with *E. calcea* represented by the German collection of molecular weight (MW) 331 (BS = 94%, BPP = 1; Figure 1). Moreover, the morphological characters of these Chinese specimens are consistent with the description of *E. calcea* (Wells, 1961). Taking *E. calcea* falling within the clade of the newly erected genus into consideration together, this species is transferred to *Alloexidiopsis*.

***Alloexidiopsis nivea*** (J.J. Li & C.L. Zhao) L.W. Zhou & S.L. Liu, *comb*. *nov*.

MycoBank: MB 844129.

*Basionym*: *Heteroradulum niveum* J.J. Li & C.L. Zhao, in Li, Zhao, and Liu, Diversity 14 (1, no. 40):5 (2022).

Notes: *Alloexidiopsis nivea* was recently described as a member of *Heteroradulum* (Li et al., 2022a). When the independence of this species was phylogenetically supported, its relationship with additional species of *Heteroradulum*, however, failed to receive reliable statistical support in the original phylogeny with a sampling on *Auriculariaceae* (Figure 1 in Li et al., 2022a). Although the original phylogeny with a narrower sampling focusing mainly on *Heteroradulum* did not reject the close relationship of *H. niveum* with other species of *Heteroradulum*, the practice for this phylogenetic analysis (lack of additional in-group taxa for reference) cannot accurately determine the monophyly of *Heteroradulum* and, thus, the phylogenetic position of *H. niveum* (Figure 2 in Li et al., 2022a). Including a broader sampling of reference sequences, the current phylogeny unambiguously recovers this species in the newly erected genus *Alloexidiopsis* (Figure 1), so we formally propose the transfer here.

**Figure 4:**
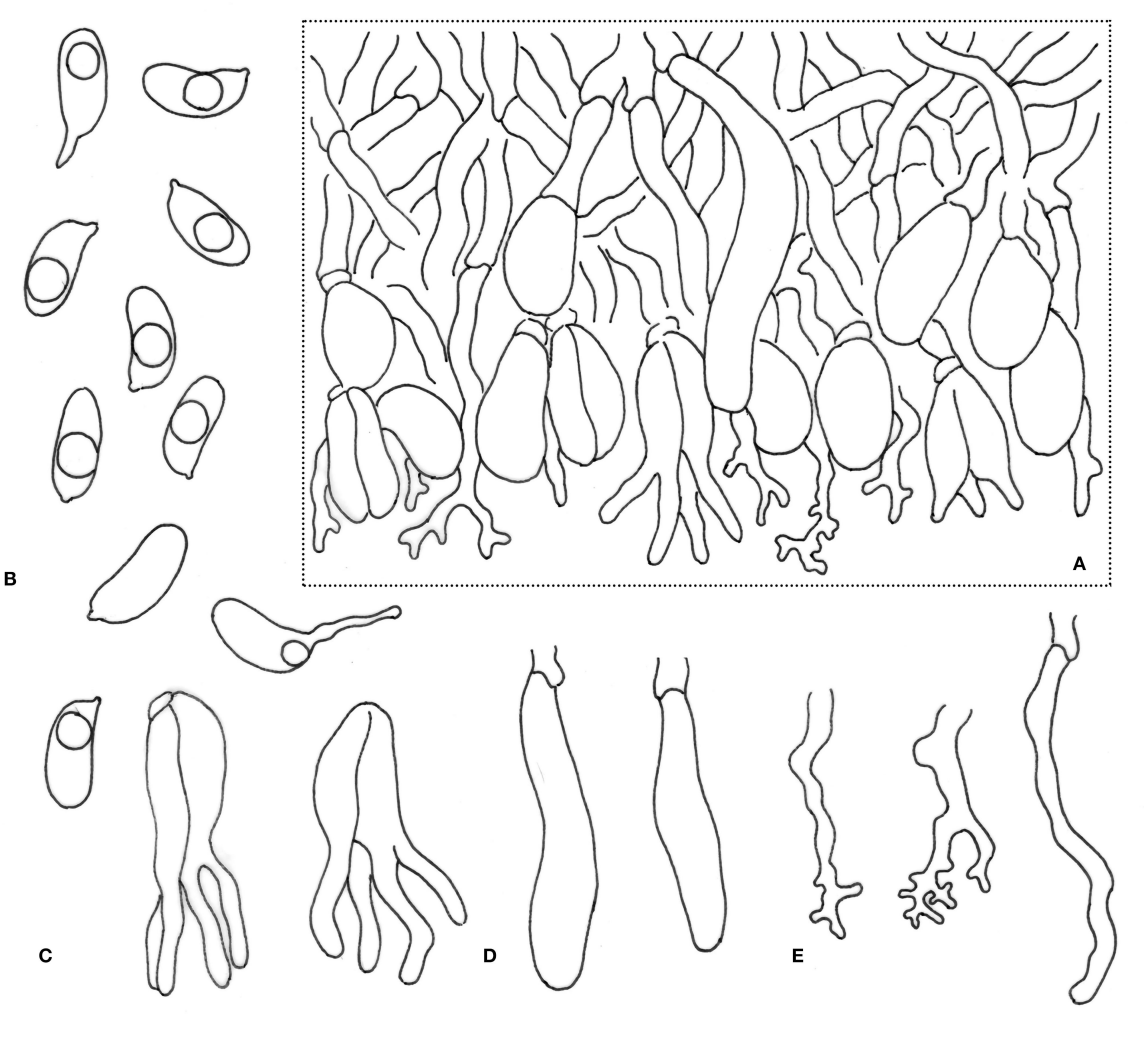
Microscopic structures of *Alloexidiopsis schistacea* (drawn from the holotype). **(A)** A section of hymenium. **(B)** Basidiospores. **(C)** Basidia. **(D)** Cystidia. **(E)** Hyphidia. Scale bars = 10 μm.

***Alloexidiopsis schistacea*** L.W. Zhou & S.L. Liu, *sp. nov*. (Figures 2E,F, 4).

MycoBank: MB 844127.

Etymology: *schistacea* (Latin), refers to the slate-like color (grayish) of hymenophore.

Diagnosis: Characterized by grayish hymenophore with small tubercles.

Type: **China:** Sichuan, Pingshan County, Laojunshan National Nature Reserve, on the fallen angiosperm trunk, 19 Aug 2020, *LW Zhou*, LWZ 20200819-21a (holotype in HMAS).

Description: Basidiomes annual, resupinate, membranaceous, becoming leathery upon drying, closely adnate, widely effused, up to 15 cm long, 2.5 cm wide, about 200 μm thick. Hymenophore smooth, covered by regularly arranged sterile spines, greyish when fresh. Margin gradually thinning out, thin, concolorous with or slightly darker than subiculum.

Hyphal system monomitic; generative hyphae with clamp connections. Subiculum composed of crystal clusters and agglutinated hyphae; subicular hyphae hyaline, thin-walled, frequently branched, closely interwoven, 2–3 μm in diam. Cystidia cylindrical with an obtuse apex, 25–50 × 4–6 μm, with a clamp connection at base. Hyphidia arising from hyphae, nodulose or branched, hyaline, thin-walled, 20–40 × 1.5–3 μm. Basidia ellipsoid to ovoid, longitudinally septate, four-celled, embedded, 15–20 × 7–10 μm. Basidiospores cylindrical to broadly cylindrical, slightly curved (allantoid), hyaline, thin-walled, smooth, acyanophilous, inamyloid, indextrinoid, with oily inclusions, (8.5−)9.5–11(−12.5) × (4.3−)4.5–5.5 μm, *L* = 10.4 μm, *W* = 5.0 μm, *Q* = 2.1 (*n* = 30/1).

Notes: *Alloexidiopsis schistacea* resembles *Alloexidiopsis yunnanensis* (transferred below) by grayish, grandinioid to odontioid hymenophore; however, the latter species differs in two- to three-celled basidia and larger basidiospores (17–24 μm × 5–8 μm; Guan et al., 2020). Micromorphologically, *Exidiopsis badia* and *E. umbrina* resemble *A. schistacea* by the presence of cystidia and hyphidia; however, these two species produce gelatinous, but not leathery basidiomes (Roberts, 2003). Moreover, *E. badia* has larger basidiospores than *A. schistacea* (13–15 μm × 5.5–6 μm; Roberts, 2003). Although only one collection is available for *A. schistacea*, its distinct morphological characters and phylogenetic position make the large enough basidiomes suitable to be described as a new species.

***Alloexidiopsis yunnanensis*** (C.L. Zhao) L.W. Zhou & S.L. Liu, *comb*. *nov*.

MycoBank: MB 844130.

*Basionym*: *Heteroradulum yunnanense* C.L. Zhao (as “*yunnanensis*”), in Guan, Liu, Zhao and Zhao, Phytotaxa 437(2):57 (2020).

Notes: *Alloexidiopsis yunnanensis* was originally described in Yunnan, China as a member of *Heteroradulum* (Guan et al., 2020). However, the generic placement of this species is inaccurate as indicated in a study by Li et al. (2022b), who, thus, excluded it from *Heteroradulum* and left its generic position open. The current phylogeny recovers this species in the newly erected genus *Alloexidiopsis* (Figure 1), so we formally propose the taxonomic transfer here.

### A Key to All the Five Species of *Alloexidiopsis*

1. Hymenophore smooth…………………………………2
2. Hymenophore grandinioid to odontioid…………………4
3. Basidiospores less than 7 μm wide…………………*A. nivea*
4. Basidiospores more than 7 μm wide……………………3
5. Hymenophore greyish white to ochraceous when fresh; in the Northern Hemisphere……………………………*A. calcea*
6. Hymenophore cream to pale orange when fresh; in the Southern Hemisphere………………………*A. australiensis*
7. Basidiospores more than 14 μm long………*A. yunnanensis*
8. Basidiospores less than 14 μm long……………*A. schistacea*

## Discussion

In this study, we further revise the generic delimitation of corticioid fungi in *Auriculariales* based on previous studies (Malysheva and Spirin, 2017; Li et al., 2022b). A new genus *Alloexidiopsis* is erected for two new species, namely, *A. australiensis* and *A. schistacea*, a new combination from *Exidiopsis* as *A. calcea* and two new combinations from *Heteroradulum* as *A. nivea* and *A. yunnanensis*. A key to all the five species currently accepted in *Alloexidiopsis* is provided.

Besides the five accepted species, two unnamed distinct lineages are recovered in *Alloexidiopsis* (Figures 1, 2G,H). The poor growth stage of these specimens restricts accurate morphological examinations, so no taxonomic treatment is proposed for them. However, this phylogeny indicates that the species diversity in *Alloexidiopsis* could be higher. Systematic field trips for collections of *Alloexidiopsis* and comprehensive taxonomic studies will result in more new members of *Alloexidiopsis*.

After the transfer of *Exidiopsis calcea* to *Alloexidiopsis, Exidiopsis* is closer to being a monophyletic genus. A sample “*E. grisea*” (TUFC100049) also falls in the clade of *Alloexidiopsis*, whereas another collection of this species (RK 162) is separated far from *Alloexidiopsis* as a basal lineage of *Auriculariaceae* (Figure 1). We have neither collection for morphological examinations and, thus, cannot challenge the taxonomic determinations given. Moreover, the texture of *E. grisea* is waxy gelatinous (Wells, 1961), which makes this species distinguished from all the members of *Alloexidiopsis*. Consequently, it is premature to change the taxonomic position of *E. grisea* at this stage.

It is noteworthy that the same research group separately described two new species of *Heteroradulum*, viz., *H. niveum* and *H. yunnanensis* quite recently (Guan et al., 2020; Li et al., 2022a). However, the generic placement of these two species is inaccurate and thus, they are transferred to the new genus *Alloexidiopsis*. Even if the inaccurate placement has mainly resulted from the practice of phylogenetic analyses, this phenomenon also indicates that the taxonomic system of *Auriculariales* is poorly established. It has not been tried to do so since the publication of Weiß and Oberwinkler (2001) 20 years ago, which even leaves the monophyly of *Auriculariales* unconfirmed. A multilocus-based phylogeny with a wider sampling of various morphological groups in *Auriculariales* is urgently needed to achieve a more natural classification of this order, as in other orders within *Agaricomycetes* (Wang et al., 2021).

## Data Availability Statement

The data presented in the study can be found in the GenBank (https://www.ncbi.nlm.nih.gov/GenBank; accession numbers: OM801918-OM801947) and TreeBASE (http://www.treebase.org; accession number: S29452) repositories.

## Author Contributions

S-LL, Z-QS, X-YL, and L-WZ made morphological examinations. S-LL and Q-ZL performed phylogenetic analyses. L-WZ conceived and supervised the study. S-LL, Z-QS, and L-WZ wrote the manuscript. All authors have approved the final version of the manuscript.

## Funding

This study was financed by the Biodiversity Survey and Assessment Project of the Ministry of Ecology and Environment, China (Project No. 2019HJ2096001006) and the National Natural Science Foundation of China (Project Nos. 31970012 and 32100004).

## Conflict of Interest

The authors declare that the research was conducted in the absence of any commercial or financial relationships that could be construed as a potential conflict of interest.

## Publisher's Note

All claims expressed in this article are solely those of the authors and do not necessarily represent those of their affiliated organizations, or those of the publisher, the editors and the reviewers. Any product that may be evaluated in this article, or claim that may be made by its manufacturer, is not guaranteed or endorsed by the publisher.
